# Ten simple rules for large-scale data processing

**DOI:** 10.1371/journal.pcbi.1009757

**Published:** 2022-02-10

**Authors:** Arkarachai Fungtammasan, Alexandra Lee, Jaclyn Taroni, Kurt Wheeler, Chen-Shan Chin, Sean Davis, Casey Greene

**Affiliations:** 1 DNAnexus, Inc., Mountain View, California, United States of America; 2 Genomics and Computational Biology Graduate Program, University of Pennsylvania, Philadelphia, Pennsylvania, United States of America; 3 Childhood Cancer Data Lab, Alex’s Lemonade Stand Foundation, Philadelphia, Pennsylvania, United States of America; 4 Center for Health AI, University of Colorado Anschutz School of Medicine, Aurora, Colorado, United States of America; 5 Department of Medicine, Divisions of Medical Oncology and Hematology, University of Colorado Anschutz School of Medicine, Aurora, Colorado, United States of America; 6 Department of Biochemistry and Molecular Genetics, University of Colorado Anschutz School of Medicine, Aurora, Colorado, United States of America

## Introduction

Exabytes of images, sequences, tabular data, and unstructured data are now available for analysis to advance science. These data support efforts to visualize the image of the black hole [1], characterize the spectrum of mutations across cancer types [2], create a sophisticated language model [3], and many other tasks. Defining a data analysis as large scale or not is likely to be a moving target as computing and data transfer technologies advance. We consider an analysis large scale when the data size exceeds the capacity of local resources or when the amount of time spent waiting for high-performance computing (HPC) compute capacity would disrupt the pace of the research project. For example, the recount2 [4] analysis processed petabytes of data, so we consider it to be large-scale data processing for the current day standard.

We provide 10 simple rules that apply whether the infrastructure used is traditional HPC or a more modern cloud service. Our work and experience are in the space of genomics, but the 10 rules we provide here are more general and broadly applicable given our definition of large-scale data analysis. The particular characteristics of genomics data that influence our recommendations but that may not be universally true are that the full set of such data does not have to be present to begin processing, that many processing steps can be completed in a streaming manner, and that the size of processed data required for downstream analysis and interpretation is typically orders of magnitude smaller than the unprocessed form.

### Rule 1: Don’t reinvent the wheel

Large-scale data processing presents engineering and scientific challenges that can be enticing to undertake. However, processing data at large scale is a substantial undertaking. During the course of the effort, researchers are likely to have to discover and contend with particular nuances of their HPC or cloud provider and also their data sources. Workflows and pipelines that work at a modest scale will not necessarily scale to the full set of data that must be processed. Throughout the entire effort, it will be important to record the provenance of data and processing steps to know that the processed data that result at the end remain comparable. In short, undertaking large-scale data processing will require substantial planning time, implementation time, and resources.

There are many data resources providing preprocessed data that may meet all or nearly all of one’s needs. For example, Recount3 [4,5], ARCHS4 [6], and refine.bio [7] provide processed transcriptomic data in various forms and processed with various tool kits. CBioPortal [1,8] provides mutation calls for many cancer studies. Cistrome provides both data and tool kit for transcription factor binding and chromatin profiling [9,10]. A research project can be substantially accelerated by starting with an existing data resource.

### Rule 2: Document everything

“If it’s not written down, it didn’t happen.”

A decision log can help keep track of the rationale behind decisions and who made them. As members join and leave the team working on a large-scale data processing project, remembering why each decision was made can become difficult. A simple log with what the decision is, what the rationale for it is, who contributed to making it, and who agreed or approved with it can be enough. This helps avoid reassessing different analysis choices, parameter configurations, data sources, or tool choices. This information can also be helpful to have consolidated when creating documentation explaining the pipelines used [11].

There are multiple approaches to logging decisions, but a powerful and common approach is to repurpose project management systems such as GitHub Issues. Doing so effectively ties decisions to code or documentation changes, allows for historical record including discussion, can handle decision dependencies, and can incorporate formal review process automation.

### Rule 3: Understand hardware and regulatory limitations and trade-offs

Selecting potential computing platforms is a multi-objective optimization process where the goal is to minimize cost—with respect to computing cost or quota, waiting time, and implementation time—while maximizing the utility of the data returned. The difference between large-scale and more traditional scale processing is that the investment in infrastructure for repeatability and selecting and rerunning certain subsets of the data becomes much more valuable as the processing task grows.

Certain projects, particularly those that include sensitive or identifiable data, are also subject to regulations that may affect hardware or computing options. Certain types of clinical data can require a computing platform to meet specific standards for data security and access control. In addition, many countries have data locality policies prohibiting data transfer out of the country. In regulated settings, researchers may need to access data through a defined computing platform and only publish certain summary-level results.

Performing computing where the data is located is also becoming popular. This model may make it easier to meet regulatory guidelines and avoids the cost and transfer time associated with moving large datasets to different locations. This model works particularly well for genomics in which the analyzed outputs are usually much smaller than the inputs.

While evaluating potential computing platforms, it is useful to examine the constraints and opportunities for optimization that these selections impose. Is computing available on a first-come-first-serve basis? Are there hard limits on resources per task or per user? How do the specific features of hardware, network, and storage impact execution time? Each of these elements should be recorded to make a realistic plan and set milestones.

### Rule 4: Automate your workflows

Automation in the form of robust pipelines described as code is a key foundation for success of large-scale data processing as it demonstrates it is technically possible to process the data without intervention. There are often many different ways to design end-to-end pipelines, so it is often helpful to perform initial experiments with multiple configurations to find the most suitable framework.

However, even with a well-crafted pipeline, for large-scale data processing, it is often not trivial to simply run such a process hundreds of thousands to millions of times without changes. As processing steps will need to be adjusted based on rare occurrences that are difficult to predict in advance, it is important to use or implement workflow systems that record key elements of data and processing so that one can programmatically rerun processing where required.

There are workflow tools that can support many use cases including workflow languages and systems such as Workflow Description Language (WDL) [12], Common Workflow Language (CWL) [13], Snakemake [14], or Nextflow [15]. The researcher could choose the options that are compatible with chosen computing platforms for both technical and compliance reasons.

### Rule 5: Design for testing

A key element of a large-scale data processing effort is a robust set of test examples that can be easily run. Events that rarely occur when processing thousands of entities are encountered frequently when processing millions. It is not possible to define a perfect test set in advance of processing data. However, one can use domain-specific expertise to identify cases that may cause problems or that may be particularly informative with respect to resource needs.

If the goal is to process sequencing data, including studies with particularly deep sequencing and particularly shallow sequencing can be wise. In sequencing data, it is possible that reads may be sorted or in arbitrary order: make sure that variable patterns that can be expected in valid input are covered by tests. If files can be derived from multiple sources, using different technologies, or are in multiple formats, include examples of each possible case. It can also be wise to have a random subsample available as a test case, as this set can help one estimate how much time and resources will be required to complete the effort. Be prepared to extend the test set as new edge cases present themselves.

It is also wise to test with invalid inputs and cases that are expected to fail such as inputs that are too large, too small, or malformed. Determining what errors are observed and using these error states to annotate inputs with unknown validity can help to triage data that fail to process. It can be particularly problematic when execution succeeds but the output is missing, empty, or truncated. Conversely, the execution could hang indefinitely or until the maximum duration allowed by the system. In all these cases, it is important to design processing steps to detect and annotate such failures.

### Rule 6: Version both software and data

Applying version control to all code is always recommended for reproducible research [16,17]. In the context of a large-scale data analysis, we need to go beyond this initial step because data processing is expected to take place over an extended period of time. The longer a project’s timeline, the more likely that a tool will be updated to include breaking changes or even become unavailable. This is of particular concern if such large-scale data analysis includes legacy data from older technologies.

To guard against interruptions, all dependencies should be pinned to a specific version (ideally through a version control hash or equivalent) that has been thoroughly tested (Rule 5). Using specific versions of dependencies can also help mitigate any technical effects that would be introduced by using different versions or configurations of the software. Utilizing container technology is highly encouraged, if allowed in the system, to guarantee the computing environment for processing. Using container technology also helps if multiple computing infrastructures are required or desired.

When multiple processing steps are combined into workflow (Rule 4), the workflow itself should be versioned. Each workflow language itself is a tool that is evolving with new syntax and functionality, so the researcher should specify which version is used and tested. The code for each configuration of the workflow should also be kept and versioned to allow for reversion to or comparison to older versions when needed.

In biomedical data analysis, there are often components beyond the software and related dependencies that need to be included in the reproducibility plan. For instance, if the data processing relies on a genome build, using the most recent build and release in the pipeline will be insufficient. Instead, the processing needs to be tied to a specific build and release, much like the dependencies in the pipeline.

Taking these steps does not mean that the pipeline cannot be updated during the study. However, such change should be conscious and taken into account during the data analysis. Any analysis pipeline should include explicit steps to capture and record all relevant software and data resource versions.

### Rule 7: Continuously measure and optimize performance

Computing at scale means a considerable resource investment. Adjustments that can reduce the time, memory, or disk usage required by a factor of 2 or even 10 may cost more in person time to optimize than they return in savings when processing data at modest scale. However, when the total computing cost of processing nears or exceeds personnel costs, the return on investment from optimizing can be substantial and justifiable.

Very simple steps include examining manuals for the software being applied to determine whether or not certain calculations or options can be disabled to save time. Matching the command line parameters, such as the number of threads for multithreaded software, to the exact computing resources available is a simple adjustment that can provide substantial savings. Careful management of files and data, removing those that are no longer required, can also save resources without fundamentally altering the initially implemented processing software. Liberal use of caching of reusable results (e.g., sequence indexes, pretrained models, or resource files that require preprocessing), a feature available in some workflow systems, can provide additional cost savings.

Depending on the scale of the task at hand, it may make sense to more fundamentally reexamine the processing steps in light of the data gathering performed through the preceding rules. Are there any other tools that would produce functionally equivalent results that have better performance characteristics? Does it make sense to run one or more steps on accelerated computing hardware including graphics processing units? If disk or transfer constraints add significantly to CPU time usage, does it make sense to use memory as intermediate storage for some steps? Many cloud providers provide low-cost instances that may be interrupted or only intermittently available, while HPCs sometimes have queues for jobs that require less wall time that allow more concurrent processes. For large efforts, it is important to consider whether taking advantage of such strategies can make the work substantially more economical to be completed.

### Rule 8: Manage compute, memory, and disk resources at scale

Unlike processing small datasets, when the datasets are large, trade-offs in memory (i.e., RAM), disk space, and processing time take on more significance.

There may be cases where the dataset is too large to be read into memory. If the execution does not have built-in memory or data streaming control, the analysis will fail due to out of memory. One solution is to partition the analysis into modules and save intermediate files to disk. However, this will cost some disk space and processing time to write to file. In machine learning, most programming frameworks offer generators or minibatch options to allow training with partial data usage. It would take more time to process the same amount of data using this approach, but this could be preferable for large datasets. Another option is to delete old variables that are storing large amounts of data such as the command “del” in Python and “rm” in R. Although both R and Python have garbage collectors, these commands can still be helpful for active memory management.

An additional consideration is if resources are shared such as the local HPC. Even if the large dataset can be loaded into memory, most of the memory in the cluster could be occupied and prevent other members from using the computing resources. However, your analyses could complete faster if you are able to load your input dataset in full versus in batches. These types of trade-offs need to be considered and communicated with other users in the institution when working with large datasets.

### Rule 9: Learn to recover from failures

Rule 5 discusses how to design tests that include both expected successes and failures. Even if a potential failure mode is not found on real production data, setting up a system to handle the unexpected is still critical. The more data you analyze, the higher chance that an unexpected or untested failure will occur. Handling failures during large-scale data processing is similar to handling failure in other computing settings.

Certain failures may be transient—for example, those that arise due to a sporadic hardware failure, which can also occur in cloud environments. In these cases, rerunning the step is often sufficient. If certain datasets require more resources, escalating resource requests can be designed for rerun jobs. The most challenging failures will be the “one in a million” settings that happen routinely when processing large amounts of data. These require manual diagnosis, so the system designer needs to consider how to handle downstream analyses that depend on this output.

In Rule 4, we discuss automating the end-to-end pipeline. Those ideas could be expanded for automated handling of failure as well. It can often be helpful to automatically schedule different resources or execution characteristics based on attributes of data such as data size. Handling predictable errors before they happen often reduces the overall workload.

If the data analysis is time sensitive and needs rapid turnaround, it’s helpful to continuously rerun and monitor end-to-end tests for failures. These test runs could reveal problems before they occur in time-sensitive data processing.

### Rule 10: Monitor execution

Even with the best planning, unexpected issues could be just around the corner. Active monitoring is the key to make sure if our assumption about data analysis is closely matched to reality. Does the individual analysis take similar time to the estimated hours? Are there a large variation of analysis hours? Does the ratio of failed analysis exceed what can be tolerated? Does the number of outputs agree with the number of finished analyses?

The importance of ongoing monitoring and visibility into workflow and analysis results cannot be overstated. Developing dashboards and maintaining databases of results can add value, but such efforts must be evaluated based on expected benefit, developer time and expertise, and whether such tooling is desirable for other reasons (collaborators or public users of data resources).

It is hard to fix an ongoing production run. We recommend scaling up processing capacity roughly an order of magnitude at a time and working in well-defined blocks of analysis. For example, if 1,000 analyses need to be done, run and monitor the first 10, then 100 before starting on 1,000. This helps to manage the total amount of time or money spent on analyses that ultimately need to be repeated. Once each set of analyses succeed or at least produces expected failures, the next order of magnitude can be approached.

## Conclusions

Large-scale data processing requires not only a substantial amount of individual work but also compute resources and other elements. It is helpful to reduce the need for others to perform the same or very similar analyses. The results of large-scale data processing can serve as impactful contributions in their own right. We recommend that the results be shared wherever possible and that metrics of reuse be part of the evaluation of success. One excellent means to do this is by preparing a publication describing the work and the resource. It is helpful to provide detailed information of how such results are generated. This includes the data source(s), inclusion and exclusion criteria, tools and versions, and a high-level intuition of the purpose for each step. Having even more detailed documentation associated, such as the specific commands run, will help in drafting the publication and also will help others to build upon the resource.

We focused primarily on the technical aspects of such research, but it is important that research be undertaken responsibly [18]. While there are many potential rules that could have been added, we highlight those that we find most salient in our own work and in the processing efforts that we have explored from others. We provide rules that are agnostic to the underlying computing platform. The optimal choices on a given platform will differ based on the cost structures of the underlying environment.
